# Supplementary material for: Patient interest in the development of a center for Ehlers-Danlos syndrome/hypermobility spectrum disorder in the ﻿Chicagoland region
Source: Orphanet J Rare Dis. 2024 Mar 14;19:122. doi: 10.1186/s13023-024-03109-w (PMC10938836; doi:10.1186/s13023-024-03109-w)
Supplement: Supplementary file 1 — Additional file 1: Utilization of medical disciplines. [file 13023_2024_3109_MOESM1_ESM.pdf]

EDS/HSD and related conditions - Center of Excellence in Chicago interest surve

Introduction and Consent to Participate

As EDS/HSD and related conditions are better understood, there is a recognized gap in high-quality health care to adequately and effectively address patients' complex medical needs. Although it exists in other areas, there is not yet a coordinated care system where patients can provide their medical input to one system and have access to a collaborative team of EDS/HSD informed health care providers. We would like to know patients' level of interest in the concept and their likelihood of accessing such a system in the future.

Thank you for agreeing to participate in our research by completing this survey. Your participation is voluntary. There are no benefits to participation except helping researchers in the future. Before you begin, please note that this survey is for individuals 18 or older, who have a diagnosis of or believe they have Ehlers-Danlos Syndrome and/or related conditions. If you do not meet these criteria, please do not complete this survey. You do not have to answer any questions that make you uncomfortable. All data will be de-identified and stored on a password-protected computer. With questions about the research, please contact Wendy Wagner at wendyw.edsil@gmail.com.

* 1. Do you agree?

- ○ Yes
- ○ No

EDS/HSD and related conditions - Center of Excellence in Chicago interest surve

Demographics

We would like to learn a little more about the community.

2. What is your age?

- Under 18
- 18-24
- 25-34
- 35-44
- 45-54
- 55-64
- 65+

3. How do you define your gender?

- Male
- Female
- Assigned male at birth, currently identify as female
- Assigned female at birth, currently identify as male
- I do not identify with male or female gender

4. Where do you live?

- In the Chicago city limits
- In the suburbs of Chicago but not in the city of Chicago
- In the state of Illinois but not in the city or suburbs of Chicago
- If other than Illinois, please list which state or country
- In a state other than Illinois
- Outside of the United States

5. What kind of health insurance do you have?

- Medicare
- Medicaid
- Private insurance (such as BCBS, Humana, Aetna, etc) ONLY
- Other (please specify)
- A combination of government (Medicare/Medicaid) and private insurance
- I do not have health insurance

6. Approximately how much have you spent on your healthcare in the past calendar year -- including insurance premiums, copays, deductible, out of pocket, medications/supplements, medical equipment?

- $0 to $999
- $1,000 to $4,999
- $5,000 to $9,999
- $10,000 to $19,999
- $20,000 or more

Your Health History

Please tell us a little more about your past and current health experiences.

7. Do you have an official diagnosis from a medical provider of any of the following conditions? Choose "**formally diagnosed**" or "**suspected**" if applicable. Select ALL that apply.

	Formally diagnosed	Suspected but not formally diagnosed	Ruled out	Not considered
Hypermobile Ehlers Danlos Syndrome (hEDS) or Hypermobility Spectrum Disorder (HSD)	◯	◯	◯	◯
Ehlers Danlos Syndrome (EDS, non-hypermobile)	◯	◯	◯	◯
Dysautonomia and/or Postural Orthostatic Tachycardia Syndrome (POTS)	◯	◯	◯	◯
Mast Cell Activation Syndrome/Disorder (MCAS)	◯	◯	◯	◯
Cranial cervical instability (CCI)	◯	◯	◯	◯
Anxiety	◯	◯	◯	◯
Depression	◯	◯	◯	◯
Migraine	◯	◯	◯	◯
Irritable Bowel (IBS)/GI distress	◯	◯	◯	◯
Temporomandibular Joint Dysfunction (TMJ)	◯	◯	◯	◯
Fibromyalgia	◯	◯	◯	◯
Arthritis	◯	◯	◯	◯

8. If you have one of the diagnoses above, what is the longest amount of time it took for you to receive that diagnosis - from onset of symptoms to official medical diagnosis? Please enter a whole number of YEARS.

[ ]

9. How much do your physical symptoms related to EDS/HSD/hypermobility and related symptoms limit your ability to work/go to school and/or participate in preferred recreational activities?

- ◯ A significant amount. I do not work/go to school and can not participate in preferred work or activity.
- ◯ A moderate amount. I am able to work/go to school and participate in preferred activities but not as much as I want/need
- ◯ Some. I am able to work/go to school and participate in preferred activities. I have symptoms but they do not limit me.
- ◯ None. I do not have symptoms.

10. Regarding medical trauma or medical gaslighting -- the experience a patient has when a medical provider discounts or minimizes symptoms or inappropriately considers them psychosocial instead of physical in origin -- please check the sentence that best describes your past experiences.

- ◯ I have never experienced medical trauma or medical gaslighting
- ◯ I have experienced medical trauma or medical gaslighting, but it does not affect my willingness to access medical care in the future.
- ◯ I have experienced medical trauma or medical gaslighting, and it makes me delay my medical care. Sometimes I avoid or put off medical appointments because I fear the potential for medical trauma or gaslighting.
- ◯ I avoid medical appointments as much as possible because the fear of having an experience of medical trauma or gaslighting is worse than the symptoms I am experiencing.

PAST AND CURRENT: Access to medical care

We would like to know how you access medical care (in the past and currently) related to your EDS/HSD and related conditions (diagnosed or not diagnosed) and related complaints.

11. As it relates to symptoms from EDS/HSD and related conditions, how satisfied are you with the care that you are currently receiving from your local medical providers?

- ◯ Very satisfied
- ◯ Satisfied
- ◯ Neither satisfied nor dissatisfied
- ◯ Dissatisfied
- ◯ Very dissatisfied

12. How easy do you find it to access quality care for your symptoms related to these conditions?

- ◯ Very easy
- ◯ Easy
- ◯ Somewhat easy
- ◯ Neither easy nor difficult
- ◯ Somewhat difficult
- ◯ Difficult
- ◯ Very difficult

13. What health care systems have you accessed in the past 5 years? Select <u>ALL</u> that apply.

- ☐ Northwestern Memorial Healthcare
- ☐ Loyola Medicine
- ☐ University of Chicago
- ☐ Northshore
- ☐ Edward/Elmhurst Health
- ☐ Advocate Health
- ☐ Other (please specify)

____

- ☐ None of the above
- ☐ Rush University
- ☐ University of Illinois - Chicago
- ☐ Amita Health
- ☐ County health services
- ☐ Cash pay private practice

14. *Approximately* how many medical providers have you seen for symptoms related to EDS and related conditions?

- ◯ 0-5
- ◯ 6-10
- ◯ 11-15
- ◯ 16+

15. What medical disciplines have you accessed looking for help for your symptoms related to EDS/HSD, dysautonomia, MCAS and related symptoms in the **PAST** 5 years? Select ALL that apply.

- ☐ Primary Care Provider/Internal Medicine/Family Medicine
- ☐ Neurology
- ☐ Cardiology
- ☐ Orthopedics
- ☐ Rheumatology
- ☐ Genetics
- ☐ Urology
- ☐ Physical Therapy
- ☐ Pelvic Floor Physical Therapy
- ☐ Occupational Therapy
- ☐ Opthalmology
- ☐ Ear Nose and Throat (ENT)
- ☐ General surgery
- ☐ Neurosurgery
- ☐ Other (please specify)
- ☐ None of the above
- ☐ Pain management
- ☐ Gastroenterology
- ☐ Psychiatry
- ☐ Psychologist/Talk therapist/Counselor
- ☐ Social worker/Case worker to help you manage your healthcare
- ☐ Obstetrics/Gynecology
- ☐ Sleep Medicine
- ☐ Dermatology
- ☐ Dietetician
- ☐ Allergy/Immunology
- ☐ "Alternative medicine" (chiropractic, acupuncture, massage, etc)
- ☐ Integrative/Functional Medicine Specialist
- ☐ Radiology/Imaging facility

16. How satisfied are you with how well your past and/or current health care providers have collaborated on your care?

- ○ Very satisfied
- ○ Satisfied
- ○ Somewhat satisfied
- ○ Neither satisfied nor dissatisfied
- ○ Somewhat dissatisfied
- ○ Dissatisfied
- ○ Very dissatisfied

17. How important to your health outcomes do you believe it is for your health care providers to collaborate on your care?

- ○ Extremely important
- ○ Very important
- ○ Somewhat important
- ○ Not so important
- ○ Not at all important

FUTURE HEALTH CARE: Level of Interest in a possible multi-disciplinary clinic in Chicago

As EDS/HSD and related conditions are better understood, we are recognizing the gap that exists in quality health care to adequately and effectively address patients' complex medical needs. Although it exists in other metropolitan areas, we do not yet have a coordinated care system or clinic where patients can provide their medical input to one system and have access to a collaborative team of health care providers. We would like to know your level of interest in the concept and your likelihood of accessing such a system in the future.

18. If a Center of Excellence for EDS/HSD and related conditions was developed in the Chicagoland area, how likely is it that you would access any services at all within the next 1-2 years?

- ◯ Very likely
- ◯ Likely
- ◯ Somewhat likely
- ◯ Neither likely nor unlikely
- ◯ Somewhat unlikely
- ◯ Unlikely
- ◯ Very unlikely

19. If you had access to a medical provider with expertise in EDS/HSD and related disorders, how likely would you try to access these specific services in the **FUTURE** -- within the next 1 to 2 years?

	Very likely	Somewhat likely	Not likely
Primary Care Provider/Internal Medicine/Family Medicine	◯	◯	◯
Neurology	◯	◯	◯
Cardiology	◯	◯	◯
Orthopedics	◯	◯	◯
Rheumatology	◯	◯	◯
Genetics	◯	◯	◯
Urology	◯	◯	◯
Physical Therapy	◯	◯	◯
Pelvic Floor Physical Therapy	◯	◯	◯
Occupational Therapy	◯	◯	◯
Opthalmology	◯	◯	◯
Ear Nose and Throat (ENT)	◯	◯	◯
General surgery	◯	◯	◯

Neurosurgery			
Pain management			
Gastroenterology			
Psychiatry			
Psychologist/Talk therapist/Counselor			
Social worker/Case worker to help you manage your healthcare			
Obstetrics/Gynecology			
Sleep Medicine			
Dermatology			
Dietetician			
Allergy/Immunology			
"Alternative medicine" (chiropractic, acupuncture, massage, etc)			
Integrative/Functional Medicine Specialist			
Radiology/Imaging facility			

Other (please specify)

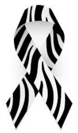

EDS/HSD and related conditions - Center of Excellence in Chicago interest surve

Preferred providers in the Chicagoland area

Are there any providers located in the Chicagoland area that you have identified that are particularly helpful and/or understand the unique needs of patients with EDS/HSD?

20. Please list their names and their corresponding subspecialties and/or institutions where they practice here:

EDS/HSD and related conditions - Center of Excellence in Chicago interest surve

Comments

21. Comments: Do you have any comments about your current or unmet healthcare needs or feedback on whether or not you believe a collaborative clinic dedicated to treating patients with EDS/HSD and related disorders would be beneficial for you? Please do not share any private medical information.

EDS/HSD and related conditions - Center of Excellence in Chicago interest surve

Thank you!

We value your time and greatly appreciate your input. THANK YOU!
